# Risk adjusted EWMA control chart based on support vector machine with application to cardiac surgery data

**DOI:** 10.1038/s41598-024-60285-2

**Published:** 2024-04-26

**Authors:** Muhammad Noor-ul-Amin, Imad Khan, Ali Rashash R. Alzahrani, Amel Ayari-Akkari, Bakhtiyar Ahmad

**Affiliations:** 1 Department of Statistics, COMSATS University Islamabad, Lahore Campus, Islamabad, Pakistan; 2 Department of Statistics, Abdul Wali Khan University Mardan, Mardan, Pakistan; 3 Mathematics Department, Faculty of Sciences, Umm Al-Qura University, Makkah, Saudi Arabia; 4 Biology Department, College of Sciences in Abha, King Khalid University, P.O. Box 960, Abha, Saudi Arabia; 5 Higher Education Department, Kabul, Afghanistan

**Keywords:** Statistical process control, Control chart, EWMA, Support vector machine, Run length, Cardiology, Health care, Mathematics and computing

## Abstract

In the current study, we demonstrate the use of a quality framework to review the process for improving the quality and safety of the patient in the health care department. The researchers paid attention to assessing the performance of the health care service, where the data is usually heterogeneous to patient’s health conditions. In our study, the support vector machine (SVM) regression model is used to handle the challenge of adjusting the risk factors attached to the patients. Further, the design of exponentially weighted moving average (EWMA) control charts is proposed based on the residuals obtained through SVM regression model. Analyzing real cardiac surgery patient data, we employed the SVM method to gauge patient condition. The resulting SVM-EWMA chart, fashioned via SVM modeling, revealed superior shift detection capabilities and demonstrated enhanced efficacy compared to the risk-adjusted EWMA control chart.

## Introduction

Statistical Process Control (SPC) is a fundamental methodology in quality management, providing a systematic framework for monitoring, analyzing, and optimizing processes. It leverages statistical techniques to ensure consistency, stability, and quality across manufacturing and service-oriented operations. The advent of Machine Learning (ML) has introduced transformative capabilities to SPC, enabling computers to learn and predict outcomes without explicit programming. ML algorithms, adept at handling large datasets, excel in identifying complex patterns and detecting subtle anomalies in real-time, augmenting traditional statistical methods. By leveraging historical and real-time data, ML algorithms forecast potential deviations, equipment failures, or defects, enabling proactive intervention. ML-driven SPC applications include predictive maintenance, anomaly detection, fault diagnosis, and process optimization, continually refining accuracy through ongoing learning. Integrating ML with SPC introduces adaptability, enhancing process monitoring and quality maintenance, thereby driving efficiency, productivity, waste reduction, and superior product quality in manufacturing and process management domains.

Walter A. Shewhart pioneered control charts in the 1920s for monitoring industrial production, evolving beyond industrial use into various fields. The standard Cumulative Sum (CUSUM) procedure is commonly used for quality monitoring but may signal changes due to patient mix variations rather than surgical performance changes. Steiner et al.^1^ pioneered the risk-adjusted control chart and introduced a new CUSUM procedure that adjusts for pre-operative patient risk, making it suitable for settings with diverse patient populations. In healthcare, adjusting for diverse patient factors, such as through the Parsonnet scoring system, aids in evaluating surgery risks by Asadyyobi and Niaki^2^. To curb false alarms, statistical studies rely on risk-adjusted control charts, crucial for accurate monitoring across diverse risk profiles. Neuburger et al.^3^ highlighted the limited adoption of statistical control charts despite clinical teams' use of time series charts for performance monitoring. Their study compared four control charts for detecting changes in rates of binary clinical data, revealing the strengths of Shewhart, EWMA, CUSUM for different rate changes, and emphasizing CUSUM's effectiveness in swiftly identifying patient safety issues causing adverse event rate increases. Zeng^4^ noted an increased emphasis on healthcare quality, highlighting the significance of monitoring care providers' performance. Utilizing continuous measures like clinical outcomes, service utilization, and cost enables prompt detection of performance changes, crucial for issue prevention and improving care quality. Zhen He et al.^5^ introduce a novel EWMA control chart for continuous surgical outcome monitoring, integrating actual survival time and predicted mortality. Simulation studies demonstrate its superior efficiency compared to existing methods, such as risk-adjusted survival time cumulative sum charts. The implementation involves individual surgeon performance monitoring based on varying patient risk levels, exemplified through a real case study. Tighkhorshid et al.^6^ utilize post-cardiac surgery survival time as a continuous quality measure, introducing a risk-adjusted EWMA control chart. Phase II evaluation using average run length criteria demonstrates improved process deviation detection, notably after integrating surgeon group effects in the regression model. Lai et al.^7^ proposed an EWMA chart for monitoring average surgical risk and variance shifts efficiently, outperforming existing cumulative sum methods in detecting variance changes and slight shifts in surgical risk. Applied to Hong Kong's Surgical Outcome Monitoring and Improvement Program data, it highlighted improvements in hospital outcomes. Asif et al.^8^ focus on the RAMA-EWMA control chart for identifying survival time after cardiac surgery, assessing its performance using a two-year dataset. Extensive simulations demonstrate its superior shift diagnostic ability compared to the control chart studied, evaluated through average run length properties (Table 1).

**Table 1:** Existence studies in literature.

S. no.	Author (year)	Type of quality characteristic	Description
1	Poloniecki et al.^9^	Binary	To detect changes in post-surgery mortality while considering variations in case mix, the study focused on binary quality characteristics monitoring during Phase II, following the Bernoulli distribution
2	Steiner et al.^10^	Binary	The procedure is exemplified using bivariate outcome data from a series of pediatric surgeries, with methodology adaptable for multivariate normal, binomial, or Poisson responses
3	Lovegrove et al.^11^	Binary	The refinement of the cumulative sum method offers a comprehensive display of surgical performance over time by accounting for each patient's risk status in assessing cardiac surgery outcomes
4	Steiner and Jones^12^	Binary	propose an updating EWMA control chart for monitoring risk-adjusted survival times in continuous time, offering ongoing estimates with favorable efficiency compared to other methods
5	Cook et al.^13^	Binary	Risk-adjusted process control charting procedures for continuous monitoring of intensive care unit outcomes, incorporating risk adjustment based on the Acute Physiology and Chronic Health Evaluation III model, are proposed as quality management tools
6	Biswas and Kalbfleisch^14^	Continuous	A risk-adjusted CUSUM procedure based on the Cox model for failure time outcomes is proposed and evaluated through simulations and application to transplant facility data from the Scientific Registry of Transplant Recipients
7	Sego et al.^15^	Continuous	A risk-adjusted survival time CUSUM chart is proposed for monitoring continuous, right-censored variables, showing higher efficiency in detecting mortality odds increases compared to the RA Bernoulli CUSUM chart, especially with low censored observations or small mortality odds increases
9	Paynabar et al.^16^	Binary	The paper introduces a risk-adjusted control chart for binary surgical outcomes, incorporating surgeon groups as categorical covariates to improve detection performance
10	Mohammadian et al.^17^	Binary	A novel risk-adjusted geometric control chart for monitoring patient survival post-surgery outperforms binary variable charts in power, demonstrated through simulations and a case study
12	Aminnayeri and Sogandi^18^	Binary	proposed risk-adjusted Bernoulli cumulative sum control charts utilize dynamic probability control limits, offering robust performance across various shifts, without assumptions about patients' risk distributions or process parameters
13	Zhang et al.^19^	Binary	apply dynamic probability control limits to risk-adjusted CUSUM charts for multiresponses, showing through simulation that their in-control performance can be tailored for different patient populations, eliminating the need for estimating or monitoring patients' risk distribution
14	Sogandi et al.^20^	Binary	This study introduces a Bernoulli state-space model with latent risk variables and dynamic probability control limits for monitoring multistage medical processes, showing satisfactory performance in identifying out-of-control stages and addressing corresponding causes
15	Asif and Noor-ul-Amin^21^	Binary	Proposed an adaptive risk-adjusted EWMA (ARAEWMA) control chart using AFT regression, outperforming traditional methods in detecting shifts in cardiac surgery patient data
16	Yeganeh et al.^22^	Binary	The paper proposes an ANN-based control chart with heuristic training for monitoring binary surgical outcomes, demonstrating superior performance compared to existing methods based on ARL, along with real-life applications and robustness analysis using the Beta distribution

Asif and Noor-ul-Amin^21^ introduce an adaptive risk-adjusted EWMA (ARAEWMA) control chart, combining AFT regression with an adaptive EWMA (AEWMA) concept. Utilizing cardiac surgery patient data assessed via the Parsonnet score method, the ARAEWMA chart demonstrates superior shift detection and efficiency compared to the risk-adjusted EWMA chart. Aslam et al.^23^ introduce upper and lower-sided improved adaptive EWMA control charts for exponential distribution-modeled data. The upper-sided chart detects upward shifts, while the lower-sided chart identifies downward shifts. Monte Carlo simulations demonstrate their superior performance over existing control charts, validated using hospital stay time data for male traumatic brain injury patients. Lai et al.^24^ introduce a GLR-based control chart for monitoring risk-adjusted ZIP processes with EWMA, demonstrating superior performance in detecting parameter shifts compared to existing methods. Application to influenza and flight delay datasets underscores its effectiveness. Rasouli et al.^25^ introduce a risk-adjusted time-variant linear state space model, applied with a group multivariate EWMA (GMEWMA) control chart to monitor multistage therapeutic processes, validated through simulation and thyroid cancer surgery, demonstrating effective real-world performance. Sogandi et al.^26^ proposed control charts, based on a Bernoulli state space model, incorporating categorical covariates and utilizing an expectation–maximization algorithm for parameter estimation, demonstrating competitive performance against Hotelling’s chart in shift detection and superiority in outlier identification, validated through simulation and real case study. Kazemi et al.^27^ proposed the RA-MTCUSUM control chart, combining AFT regression, Tukey's control chart, and multivariate CUSUM, showing robustness in simulation experiments across various distributions and real sepsis patient datasets from a Tehran hospital compared to traditional control charts. Tang and Gan^28^ develop and study a risk-adjusted EWMA charting method based on multiple outcomes, demonstrating its performance and comparability with risk-adjusted CUSUM using real surgical data. The study emphasizes the attractiveness of the risk-adjusted EWMA procedure owing to its performance and interpretability. Yeganeh et al.^22^ presents an ANN-based control chart for monitoring binary surgical outcomes, outperforming existing studies via ARL criterion. It explores machine learning in health-care monitoring, offering real-life applications, and assesses robustness by incorporating Beta distribution for mortality rates. Rafiei and Asadzadeh^29^ develop a risk-adjusted CUSUM chart for detecting declining shifts in post-surgery patient survival times, showing superior performance via a multi-objective economic-statistical model, validated in a cardiac surgery center, surpassing alternative designs in statistical and economic aspects.

Upon reviewing the related literature, it was found that all existing risk-adjusted control charts for monitoring binary surgical outcomes rely heavily on statistical assumptions. To our knowledge, there have been no studies that have employed machine learning techniques within a control chart framework for this purpose. However, the use of machine learning schemes such as support vector regression, SVM, and artificial neural network (ANN) is well-established in other process monitoring situations. To address this gap in healthcare applications, this paper introduces an SVM-based control chart (SVM-EWMA) to monitor the performance of binary surgical outcomes in Phase II. "Risk adjusted EWMA control chart" section of the paper concentrates on discussing the principles machine learning and SVM, while "Proposed SVM based risk adjusted control chart" section extensively outlines the development process and structure of the SVM-EWMA control chart. Furthermore, the performance of the newly proposed chart by utilizing run-length profiles is presented in the same section. Moving forward, “Main findings” section provides the main findings of suggested control charts, offering insights into their respective strengths and limitations. “Conclusion” section encapsulates the conclusive remarks and the overall implications of the study's findings.

## Risk adjusted EWMA control chart

In this section, we presented the RAEWMA control chart. Fit the AFT model as the first step of the design of the control chart as given in Eq. (1).

1 $$Y_{t} = \log (T_{t} ) = \beta_{0} + \beta_{1} P_{t} + \sigma \varepsilon_{t} ,$$

where *P*_*t*_ is the personnet score of the *t*th patient, $\beta_{0}$, $\beta_{1}$ are the regression coefficients, *σ* is standard deviation and ε_t_ is the random error of survival time distribution. The estimated values of $\beta_{0}$, $\beta_{1}$ and *σ* are obtained by in-control data set. where *P*_*t*_ represents the personnet score of the *t*th patient, $\beta_{0}$*, *$\beta_{1}$ are the two regression coefficients, *σ* is standard deviation and ε_t_ is the error term of survival time distribution. The values of $\beta_{0}$ = 5.07026, $\beta_{1}$ = − 0.03348 and *σ* = 0.57 are estimated by using the in-control data set as reported by Asif and Noor-ul-Amin^21^.

From the study of Tighkhorshid et al.^6^, the statistic of RAEWMA control chart is constructed by using the standardized residual (SR-AFT) from the AFT regression model. The SR-AFT is denoted by $w_{t}$ and calculated as

2 $$e_{t} = \frac{{\log (T_{t} ) - \sum\limits_{n = 0}^{n} {\beta_{n} P_{tn} } }}{\sigma },$$

3 $$z_{t} = \, \lambda e_{t}+ \, \left( {1 \, {-} \, \lambda } \right) \, z_{t - 1} ,$$

where *t* is the sample number, the *λ* is a smoothing constaa sample of size one from the residuals obtainednt range from *0* < *λ* ≤ *1*. The *z*_*o*_ is the initial value. For the improvement of the process, it is important to observe any decrease in survival time so one-sided statistic used for the RAEWMA control chart that is given by

4 $$z_{t} = \min \left\{ {\lambda e_{t} + \left( {1 - \lambda } \right)z_{t - 1} ,\xi } \right\},$$

$\xi$ indicates the mean of SR-AFT and *λ* is taken as 0.2. The variance of this statistic is given as

5 $$\sigma_{z}^{2} = \sigma_{e}^{2} \frac{{(1 - (1 - \lambda )^{2t} )\lambda }}{2 - \lambda },$$

and the lower control limit is given by

6 $$LCL_{{}} = \mu_{e} - L\sigma_{e} \sqrt {\frac{\lambda }{2 - \lambda }(1 - (1 - \lambda )^{2t} )} ,$$

where *L* is the control coefficient. The *λ* determines the decline of the rate of weights, so both of these two parameters *L* and *λ* define the in-control *ARL* to evaluate the performance of the RAEWMA chart. The similar design is adopted by Asif and Noor-ul-Amin^21^ to proposed ARAEWMA control chart.

## Machine learning and SVM

Machine learning, a groundbreaking facet of artificial intelligence, empowers computers to learn autonomously without explicit programming. It hinges on crafting algorithms enabling systems to learn from data, making predictions or decisions. Three core types exist: supervised learning, training models on labeled data to predict new outputs; unsupervised learning, detecting patterns in unlabeled data; and reinforcement learning, where agents learn via trial and error. Widely applicable across fields like finance, healthcare, and autonomous vehicles, ML extracts insights, identifies patterns, and drives efficiency. Its implementation involves data collection, preprocessing, model training, evaluation, and deployment. With technological strides, ML continues evolving, enabling computers to handle intricate tasks, shaping the landscape of intelligent systems.

SVMs are robust tools in supervised learning, adept at both classification and regression tasks by crafting optimal hyperplanes to segregate data points or predict continuous outcomes. These models emphasize maximizing the margin between distinct classes, depicted visually as a line in two dimensions or a hyperplane in higher dimensions. The crucial support vectors, positioned closest to this boundary, influence its placement and orientation. SVMs excel in handling linear and non-linear data through diverse kernel functions that transform input spaces to enable linear separability. Their strength lies in generalizing well to new data and managing high-dimensional spaces, but their performance can be sensitive to kernel and parameter choices, posing challenges with larger datasets due to computational complexities. Nonetheless, SVMs find extensive use in text classification, image recognition, and biology due to their adaptability in handling intricate decision boundaries in machine learning. Introduced by Vapnik, and Smola^30^, SVMs leverage structural risk minimization principles, surpassing empirical risk minimization of traditional neural networks. Initially designed for classification, SVMs have expanded to regression problems, demonstrating prowess in identifying optimal hyperplanes to maximize margins between classes, offering pivotal solutions for various real-world applications.

Consider the challenge of distinguishing between observations within a dataset that fall into two distinct categories. These categories are identified by labels of either − 1 or + 1. Essentially,

7 $$P = \left\{ {\left( {x^{i} ,y^{i} } \right)|x \in R^{n} ,y \in \left( { - 1, + 1} \right)} \right\}$$

Consider a hyperplane described as

8 $$\left\langle {\omega ,x} \right\rangle + b = 0$$

The hyperplane achieves optimal separation when observations are error-free and the closest vectors to it maximize their distance. Equation (7) is transformed into a canonical form Eq. (8), constraining parameters *w* and *b* accordingly.

9 $$\min_{i} \left| {\left\langle {\omega ,x^{i} } \right\rangle + b} \right| = 1.$$

In simpler terms, it asserts that the norm of the weight vector must be equivalent to the inverse of the distance from the nearest point in the dataset to the hyperplane Gunn^31^. The separating hyperplane's canonical form must adhere to this particular constraint.

10 $$y^{i} \left[ {\left\langle {\omega ,x^{i} } \right\rangle + b} \right] \ge 1,i \, = 1, \, 2, \, \ldots , \, l.$$

The distance *d*(*w*, *b*; *x*) of a point *x* from the hyper plane (*x*, *b*) is

11 $$d\left( {\omega ,b;x} \right) = \frac{{\left| {\left\langle {\omega ,x^{i} } \right\rangle + b} \right|}}{\left\| \omega \right\|}$$

Maximizing the margin q(x, b) within the limitations outlined in Eq. (10) is crucial for attaining the optimal hyperplane. This margin, defined in Eq. (4), determines the objective.

12 $$\begin{aligned} \rho \left( {\omega ,b} \right) & = \min d\left( {\omega ,b;x^{i} } \right)_{{x^{i} .y^{i} = - 1}} + \min d\left( {\omega ,b;x^{i} } \right)_{{x^{i} .y^{i} = 1}} \hfill \\ & = \min \frac{{\left| {\left\langle {\omega ,x^{i} } \right\rangle + b} \right|}}{\left\| \omega \right\|}_{{x^{i} .y^{i} = - 1}} + \min \frac{{\left| {\left\langle {\omega ,x^{i} } \right\rangle + b} \right|}}{\left\| \omega \right\|}_{{x^{i} .y^{i} = 1}} \hfill \\ &= \frac{1}{\left\| \omega \right\|}\left( {\min \left| {\left\langle {\omega ,x^{i} } \right\rangle + b} \right|_{{x^{i} .y^{i} = - 1}} + \min \left| {\left\langle {\omega ,x^{i} } \right\rangle + b} \right|_{{x^{i} .y^{i} = 1}} } \right) = \frac{2}{\left\| \omega \right\|} \hfill \\ \end{aligned}$$

Given Eq. (12), it is evident that the optimal hyperplane is the one that minimizes.

13 $$\varphi \left( \omega \right) = \frac{1}{2}\left\| \omega \right\|^{2}$$

It's important to highlight that minimizing Eq. (12) is tantamount to applying the principles of structural risk minimization (SRM). To implement SRM, it is assumed that a bound-holds, as indicated by ||x||< A. Subsequently, considering Eqs. (10) and (11), we obtain:

14 $$d\left( {\omega ,b;x} \right) \ge A$$

From the equations mentioned earlier, it's clear that the hyperplanes cannot be closer than 1/A to any data point, thereby narrowing down the feasible hyperplanes. A solution to the optimization problem in Eq. (13) under the constraint in Eq. (10) can be determined by identifying the saddle point of the Lagrange functional, as proposed by Minoux^32^.

15 $$\varphi \left( {\omega ,b,\alpha } \right) = \frac{1}{2}\left\| \omega \right\|^{2} - \sum\limits_{i = 1}^{l} {\alpha_{i} \left( {y^{i} \left[ {\left\langle {\omega ,x^{i} } \right\rangle + b} \right] - 1} \right)}$$

Involving $\alpha_{i}$ as a Lagrange Multiplier, the Lagrangian equation requires minimization concerning x and b while necessitating maximization concerning a (where a ≥ 0). Opting to solve the dual problem proves to be a more straightforward approach.

16 $$MaxW\left( \alpha \right)_{\alpha } = \max \left( {\min \varphi \left( {\omega ,b,\alpha } \right)_{\omega ,b} } \right)_{\alpha }$$

The minimum in Eq. (16) is obtained by solving the following two equations.

$$\begin{aligned} \frac{\partial \varphi }{{\partial b}} & = 0 \Rightarrow \sum\limits_{i = 1}^{l} {\alpha_{i} y_{i} = 0} \hfill \\ \frac{\partial \varphi }{{\partial \omega }} & = 0 \Rightarrow \omega = \sum\limits_{i = 1}^{l} {\alpha_{i} y_{i} x_{i} } \hfill \\ \end{aligned}$$

Replacing these equations, the solution to the problem is given by,

17 $$\begin{aligned} &\alpha^{*} = \arg \min_{\alpha } \frac{1}{2}\sum\limits_{i = 1}^{l} {\sum\limits_{i = 1}^{l} {\alpha_{i} \alpha_{j} } } y_{i} y_{j} \left\langle {x_{i} ,x_{j} } \right\rangle - \sum\limits_{i = 1}^{l} {\alpha_{k} } \hfill \\ & \alpha_{i} \ge 0,i = 1,...,l \hfill \\ & \sum\limits_{i = 1}^{l} {\alpha_{i} y_{i} = 0} \hfill \\ \end{aligned}$$

By solving these equations while accounting for the mentioned constraints, one can ascertain the Lagrange multipliers. These multipliers serve as the basis for deriving the separating hyperplane.

18 $$\begin{gathered} \omega^{*} = \sum\limits_{i = 1}^{l} {\alpha_{i} y_{i} x_{i} } \hfill \\ b^{*} = - \frac{1}{2}\left( {\omega^{*} ,x_{r} + x_{s} } \right) \hfill \\ \end{gathered}$$

where $x_{r}$ and $x_{s}$ are support vectors from each class.

## Proposed SVM based risk adjusted control chart

In this section, we introduce the proposed risk-adjusted control chart, termed RAEWMA-SVM, which utilizes SVM. While Stiner et al.^1^ utilized a logistic model for their risk-adjusted control chart, our approach, as detailed in "Risk adjusted EWMA control chart" section, is based on residuals derived from the Accelerated Failure Time (AFT) regression model by Tighkhorshid et al.^6^. In our proposed design, these residuals are obtained through the implementation of an SVM regression model. Following the methodology outlined by Tighkhorshid et al.^6^, the statistic for the RAEWMA control chart is constructed using the Standardized Residuals from the SVM regression model, denoted as SR-SVM. The RAEWMA statistic based on SR-SVM is expressed as follows:

19 $$E_{t} = \lambda v_{t} + \left( {1 - \lambda } \right)E_{t - 1}$$

In the provided formula, where *t* represents the sample number, *λ* denotes the smoothing constant within the range *0* < *λ* ≤ *1*, and $E_{0}$ is the initial value. To enhance the process monitoring, it is crucial to detect any decrease in survival time. Consequently, a one-sided statistic is employed for the RAEWMA-SVM control chart, expressed as

20 $$F_{t} = \min \left\{ {\lambda v_{t} + \left( {1 - \lambda } \right)F_{t - 1} ,\xi } \right\},$$

$\xi$ indicates the mean of SR-SVM and *λ* is taken as 0.1 and 0.25. In RAEWMA-SVM control chart, if the plotting statistic $\left|{F}_{t}\right|$ < *L*, then the process prompted the out-of-control signal.

The dataset utilized in this study is sourced from the work of Steiner et al.^1^ and pertains to patients undergoing cardiac surgery. The primary focus of analysis revolves around the survival time post-cardiac surgery, which serves as a key quality characteristic in assessing patient outcomes. In this dataset, patient information is tracked for a period of 30 days following surgery. If a patient survives beyond this interval or if there is no recorded information regarding the patient demise within the specified timeframe, the observations are deemed as right-censored. Simulation studies are conducted to evaluate the proposed approach, taking into account both patient health states and surgeon groups. A crucial risk factor considered in the model is the parsonnet score, which serves as an indicator of the patient's health state preceding cardiac surgery. This score incorporates various patient attributes such as age, gender, diabetes status, among others, to quantify the overall risk profile. The parsonnet score ranges from 0 to 100, with higher scores indicating an elevated risk of mortality post-surgery. The analysis integrates the parsonnet score as a key covariate in the model, enabling the evaluation of its impact on patient outcomes. By leveraging this comprehensive dataset and accounting for patient health states and surgeon groups, the study aims to provide insights into the effectiveness of the proposed approach in assessing survival outcomes following cardiac surgery. Control charts play a pivotal role in quality control by evaluating process performance. Their effectiveness is gauged through run length profiles, primarily focusing on two critical indicators: ARL and the SDRL, and in this study we also using the percentiles for the run length. ARL signifies the average duration data remains within control limits before detecting the first anomaly, while SDRL measures the variability in these durations. Lower values in both metrics denote superior control chart performance, indicating quicker detection of deviations from normalcy. Various computation methods—such as the Markov chain, integral equation, and Monte-Carlo simulation—exist in the literature for calculating ARLs and SDRLs. In our research, we've specifically employed the Monte-Carlo simulation technique to meticulously examine these run-length profiles. To enhance our model's accuracy, we've set fixed ARL_0_ values at 370 and 500, deliberately adjusting them across diverse shift sizes. This deliberate variation enables a comprehensive exploration that how different magnitudes of shifts influence the control chart's sensitivity and overall performance. In this research, we used the Monte-Carlo simulation technique to assess the run-length profiles by using the R language. The following steps are used to compute the run length profiles in the form ARLs and SDRLs.

**Figure 1:**
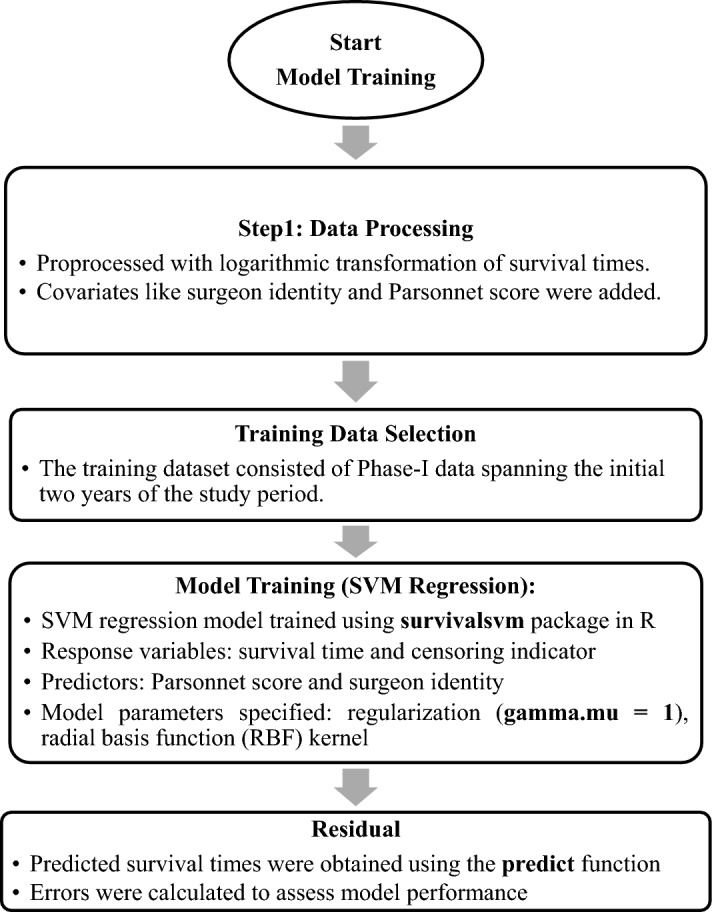
Flow chart for the suggested control chart.
Step 2: Setting up control limits
  i. Select a sample of size one from the residuals obtained in Step 1.
  ii. Using the Eqs. (19) and (20), we computed the statistic for the newly proposed control chart. This allowed us to evaluate the process's performance based on the chart's design.
  iii. We iterated the previous two steps until the process was confirmed to be within control.
  iv. If the process was found to be out of control during any iteration, we recorded the count of in-control occurrences as the run length.
  v. To calculate the in-control ARL (ARL_0_), we repeated steps (i-iii) for a total of 50,000 iterations.
  vi. If the target ARL_0_ was not achieved, we revisited the preceding steps (i-iv), this time using a different value for the control limit parameter, ℎ. This adjustment aimed to bring the process closer to the desired ARL_0_ value.
Step 3: For the out-of-control ARLs
  i. To assess the robustness of the proposed method, the out-of-control performance is examined under various shifts in the residuals at different change points. In this analysis, it is assumed that the survival time, Parsonnet score, and surgeon groups are known for each patient. Different shifts are applied to the residuals (i.e. $e_{t} + \delta$) of the SVM model, allowing for the evaluation of the method's performance under varying scenarios.
  ii. Compute the value of ARL and SDRL by repeating the process 50,000 times.

Step 1: Computing the values of residuals
  Data selection:
  i. We collected data from cardiac surgery patients, as reported in the study by Steiner et al.^19^.
  In-control dataset selection:
  i. We partitioned the dataset into two segments, using the first two years' data as the in-control dataset.
  ii. This approach aligns with the methodology outlined by Tighkhorshid et al.^6^, who also utilized the same dataset in their study.
  Residual computation:
  i. To obtain the value of et, utilized SVM model with the in-control data set for computation. The reader may consult the supplementary Appendix A for the detailed steps of SVM model fitting. The flowchart in Fig. 1 is also helpful to understand this step.
  ii. Utilizing the trained SVM model, we obtained the residual values for further analysis.

The ARL, SDRL and percentiles values for an in-control process are set at 370 and 500. The shift denoted by $\delta$. The values for shift are selected as 0.0, 0.01, 0.02, 0.03, 0.04, 0.05, 0.06, 0.07, 0.08, 0.09, 0.10, 0.40, 1.00 and 2.00. The smoothing constant is set to $\lambda$ = 0.10 and 0.25, and the outcomes are displayed in Tables 2, 3, 4, 5. Comparative results of the proposed SVM-EWMA with risk adjusted EWMA control chart for various shifts for fixed for ARL_0_ = 200 and 370 are demonstrated in Table 6.

**Figure 2:**
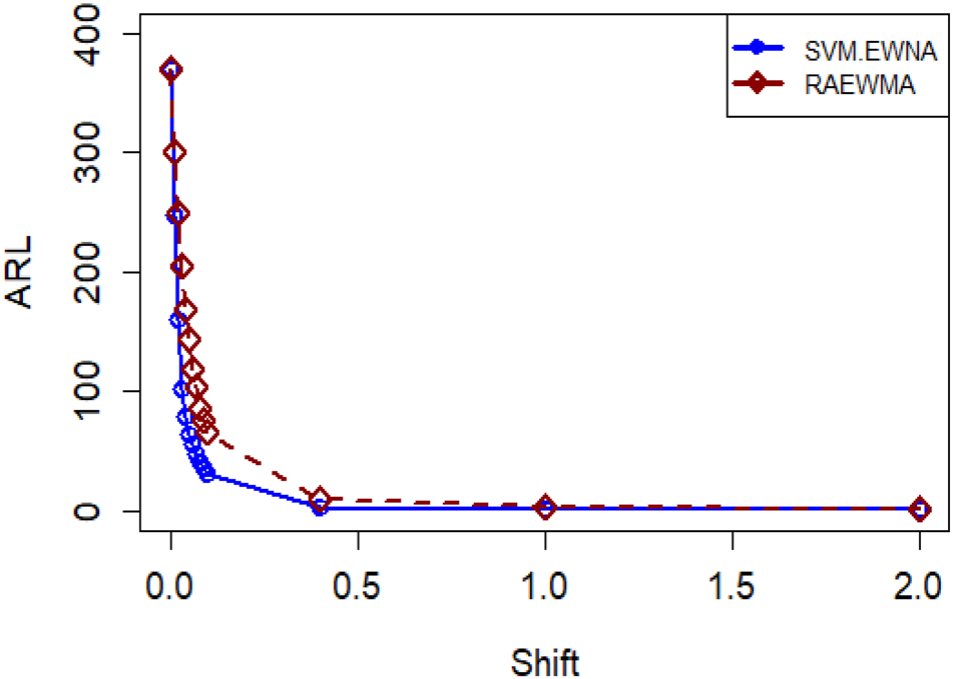
ARL plot of the suggested SVM-EWMA and RAEWMA at $\lambda$ = 0.10.

Table 2 presents the outcomes of the proposed SVM-EWMA control chart, utilizing a smoothing constant for ARL_0_ = 370 across various shifts. The smoothing constant $\lambda$ is set at 0.10. A clear trend emerges where the resulting ARL values decrease as the shift values increase, demonstrating an unbiased characteristic of the ARL. For instance, considering different shift values, such as 370.24, 250.11, 157.92, 101.13, 75.90, 65.07, 57.62, 47.69, 40.64, 35.26, 30.77, 2.92, 1.00, and 1.00, a pattern emerges where increasing shift values correspond to smaller ARL values. Figure 1 shows the flow chart for proposed SVM-EWMA control chart. Similarly, Table 3 illustrates this pattern for a different smoothing constant ($\lambda$ = 0.25) at ARL_0_ = 500. The sequence of ARL values at various shifts of 1 with a fixed shift of follows a similar trend: 370.76, 195.62, 135.28, 121.95, 104.12, 84.76, 75.74, 70.03, 63.86, 64.76, 61.28, 59.00, 1.00, and 1.00. As the shift values increase, the ARL values consistently decrease, maintaining this pattern across the shifts. Tables 3 and 5 present a comprehensive overview for ARL_0_ = 500, demonstrating the application of the recommended design for the SVM-EWMA control chart with two distinct values (0.10 and 0.25) for the smoothing constant ρ across various shifts. They illustrate the performance of the SVM-EWMA control chart under these conditions. Similarly, Table 6 provides insights into the ARL and SDRL values for both the risk-adjusted EWMA and the suggested SVM-EWMA control chart, set at ARL_0_ = 370. This table explores the impact of different smoothing constant values (0.10 and 0.25) at various shifts on the resulting ARL. Notably, the outcomes indicate that a smaller value for the smoothing constant corresponds to a smaller ARL, highlighting this relationship between the smoothing constant and resulting ARL values. This section revolves around comparing the proposed SVM-EWMA with the RAEWMA control chart, as presented in Table 6. The aim is to evaluate the SVM-EWMA's performance against the RAEWMA when ARL_0_ is set at 370, considering different smoothing constant values (0.10 and 0.25) and employing various shifts. Observing Table 6, it becomes evident that the ARL values obtained from the proposed SVM-EWMA control chart are consistently smaller compared to those derived from the RAEWMA control chart across different shift values. For instance, consider shift 0.03 with ARL_0_ = 370 and a smoothing constant of 0.10: the suggested control chart yields a value of 102.39, while the RAEWMA control chart provides a value of 204.31. This comparison reveals that the proposed model consistently generates smaller ARL values than the existing model. Similarly, at shift 0.07 for ARL_0_ = 370, the ARL value for the proposed control chart stands at 47.49, whereas for the existing control chart, it's 104.15. Figure 2 shows the significant performance of the suggested SVM-EWMA control chart compared to the RAEWMA control chart. Additionally, from Fig. 2, we can observe minor differences between the existing method and the proposed method at low and high shifts, while our proposed method efficiently performs at moderate shifts. These outcomes strongly indicate that the proposed SVM-EWMA control chart generates more efficient and superior results compared to the considered control chart, consistently showcasing its efficacy across various shift scenarios. Note that the residuals are derived from the in-control dataset using the SVM model. To create a shifted dataset, the value of the shift is added to the residuals. Subsequently, these shifted residuals are employed to assess the performance of the control charts. A similar procedure is applied for the AFT model, as outlined in Table 6.

## Main findings

This investigation serves to underscore the adeptness of our proposed chart, in swiftly and effectively identifying subtle shifts within processes, outperforming the established SVM-EWMA control chart. Through an in-depth computational analysis showcased in Tables 2, 3, 4, 5, 6, the superiority of our proposed control chart becomes distinctly evident, while Table 1 consists of existence studies. By meticulously calculating ARL and SDRL values for ARL_0_ = 370 and 500 across an array of shifts while employing smoothing constants $\lambda$ = 0.10 and 0.25, this research endeavors to shed light on the crucial insights derived from the attained results:

- Table 2 displays the outcomes obtained from employing the proposed SVM-EWMA control chart, utilizing a smoothing constant for ARL_0_ = 370 across varying shifts. A smoothing constant of $\lambda$ = 0.1 is used. The observed trend reveals a descending order in the resulting ARL values as the shift values increase, signifying the unbiased nature of the ARL. For instance, consider the ARL values corresponding to different shifts: 370.24, 250.11, 157.92, 101.13, 75.90, 65.07, 57.62, 47.69, 40.64, 35.26, 30.77, 2.92, 1.00, and 1.00. As the shift values increase, there is a consistent reduction in the ARL values. Similarly, Table 3 demonstrates a similar pattern when utilizing a smoothing constant of $\lambda$ = 0.25 at ARL_0_ = 370. The sequence of ARL values across different shifts follows a decreasing trend: 370.01, 195.62, 135.28, 121.95, 104.12, 84.76, 75.74, 70.03, 63.86, 61.76, 59.00, and 1.00. This consistent pattern further underscores that higher shift values correspond to smaller ARL values, reaffirming the trend observed in Table 1.
- Tables 4 and 5 provide a comprehensive overview focusing on ARL_0_ = 500. These tables encompass the application of the suggested design for the SVM-EWMA control chart, utilizing two distinct smoothing constant values, 0.1 and 0.3, across various shifts. A notable observation gleaned from the outcomes is that smaller values for the smoothing constant consistently yield smaller ARL values. This trend is evident across the results presented in these tables, indicating that lower values for the smoothing constant are associated with reduced ARL values, regardless of the shifts applied in the analysis.
- The comparison table, Table 6, serves to evaluate the efficacy of the proposed SVM-EWMA control chart against the corresponding risk-adjusted EWMA control chart. Both charts are assessed using smoothing constants of $\lambda$ = 0.10 and 0.25, specifically for ARL_0_ = 370 across different shifts. The findings depicted in Table 5 vividly illustrate the superior performance of the proposed SVM-EWMA control chart in yielding smaller and more efficient results compared to the risk-adjusted EWMA control chart. For example, at ARL_0_ = 370 with a smoothing constant of 0.25 and shift 0.03, the resulting ARL for the proposed SVM-EWMA and the risk-adjusted EWMA control chart are 121.95 and 292.93, respectively. A similar trend is evident at ARL_0_ = 370 with a shift of 0.08, displaying values of 63.86 for the proposed SVM-EWMA and 196.40 for the existing chart. These consistent observations affirm that the newly proposed SVM-EWMA control chart consistently outperforms the considered RAEWMA control chart.

**Table 2:** Run length and percentiles (P) outcomes of suggested SVM-based control chart at *ARL*_0_ = 370 with $\lambda$ = 0.10.

Shift	ARL	SDRL	P_05_	P_10_	P_25_	P_50_	P_75_	P_90_	P_95_
0.00	370.24	365.95	22.00	41.00	107.00	255.00	508.00	853.30	1109.05
0.01	250.11	247.43	18.00	31.00	77.00	173.00	341.25	570.00	739.00
0.02	157.92	153.48	15.00	23.00	51.00	110.00	214.00	354.00	466.00
0.03	101.13	96.12	10.00	16.00	33.00	72.00	137.25	225.00	293.00
0.04	75.90	68.30	8.00	13.00	27.00	55.00	105.00	165.00	216.00
0.05	65.07	60.36	7.00	11.00	23.00	47.00	87.00	142.00	187.3
0.06	57.62	51.66	6.00	10.00	21.00	43.00	78.00	124.00	159.95
0.07	47.69	40.67	6.00	9.00	19.00	36.00	64.00	101.00	128.00
0.08	40.64	33.44	6.00	9.00	17.00	31.00	55.00	85.00	107.00
0.09	35.26	28.17	5.00	8.00	15.00	28.00	47.00	72.00	90.00
0.10	30.77	23.28	5.00	8.00	14.00	25.00	41.00	61.00	76.00
0.40	2.92	0.31	2.00	3.00	3.00	3.00	3.00	3.00	3.00
1.00	1.00	0.00	1.00	1.00	1.00	1.00	1.00	1.00	1.00
2.00	1.00	0.00	1.00	1.00	1.00	1.00	1.00	1.00	1.00

**Table 3:** ARL and SDRL for the recommended SVM-based control chart at *ARL*_0_ = 370 with $\lambda$ = 0.25.

Shift	ARL	SDRL	P_05_	P_10_	P_25_	P_50_	P_75_	P_90_	P_95_
0.00	370.01	369.13	18.00	38.00	103.00	253.00	519.00	870.00	1117.05
0.01	195.62	193.49	11.00	21.00	56.00	135.00	274.00	452.00	581.00
0.02	135.28	133.99	8.00	16.00	39.00	94.00	188.00	307.00	402.00
0.03	121.95	118.82	8.00	15.00	37.75	86.00	167.00	276.00	360.05
0.04	104.12	100.73	7.00	13.00	32.00	74.00	144.00	235.00	303.00
0.05	84.76	82.26	7.00	11.00	26.00	60.00	117.00	191.01	250.00
0.06	75.74	74.15	6.00	10.00	23.00	54.00	103.00	174.01	224.00
0.07	70.03	67.46	5.00	9.00	22.00	49.00	97.00	158.01	204.00
0.08	63.86	61.48	5.00	8.00	20.00	45.00	88.00	143.01	166.00
0.09	64.76	62.35	5.00	8.00	20.00	45.00	89.00	146.01	190.00
0.10	61.28	59.57	5.00	8.00	19.00	43.00	83.00	139.01	183.00
0.40	59.00	57.33	4.00	8.00	18.00	41.00	81.00	135.01	173.00
1.00	1.00	0.00	1.00	1.00	1.00	1.00	1.00	1.00	1.00
2.00	1.00	0.00	1.00	1.00	1.00	1.00	1.00	1.00	1.00

**Table 4:** Run length outcomes of suggested SVM-based control chart at *ARL*_0_ = 500 with $\lambda$ = 0.10.

Shift	ARL	SDRL	P_05_	P_10_	P_25_	P_50_	P_75_	P_90_	P_95_
0.00	500.48	497.89	28.95	54.00	145.00	348.00	693.00	1159.20	1519.00
0.01	338.26	330.71	23.00	39.00	97.00	239.00	466.00	777.50	1022.25
0.02	233.71	230.11	19.00	32.00	72.00	162.00	318.00	532.80	702.00
0.03	141.23	135.27	16.00	24.00	47.00	101.00	192.00	309.70	392.35
0.04	99.68	89.42	12.00	18.00	35.00	73.00	135.00	220.00	279.00
0.05	77.61	70.37	10.00	15.00	28.00	57.00	104.00	168.00	215.00
0.06	64.05	56.32	9.00	13.00	24.00	48.00	84.00	137.00	178.00
0.07	57.08	49.86	8.00	12.00	22.00	42.00	72.00	121.00	157.00
0.08	46.80	38.62	7.00	11.00	20.00	36.00	62.00	96.00	124.00
0.09	40.05	32.54	6.00	9.00	17.00	31.00	53.00	83.00	104.00
0.10	35.51	27.62	6.00	9.00	16.00	28.00	47.00	71.00	90.00
0.40	2.92	0.31	2.00	3.00	3.00	3.00	3.00	3.00	3.00
1.00	1.06	0.25	1.00	2.00	2.00	2.00	2.00	2.00	2.00
2.00	1.00	0.00	1.00	1.00	1.00	1.00	1.00	1.00	1.00

**Table 5:** Run length outcomes of suggested SVM-based control chart at *ARL*_0_ = 500 with $\lambda$ = 0.25.

Shift	ARL	SDRL	P_05_	P_10_	P_25_	P_50_	P_75_	P_90_	P_95_
0.00	500.88	497.20	27.00	55.00	146.00	348.00	699.00	1149.00	1522.00
0.01	359.56	362.20	21.00	41.00	106.00	244.00	497.25	832.00	1089.00
0.02	198.35	197.41	13.00	23.00	58.00	138.00	276.00	459.00	576.05
0.03	134.93	129.15	9.00	17.00	42.00	97.00	183.00	307.00	394.01
0.04	122.42	122.39	8.00	15.00	37.00	85.00	164.00	280.00	364.00
0.05	102.86	100.16	7.00	13.00	31.00	71.00	144.00	238.00	306.00
0.06	83.67	80.80	6.00	11.00	26.00	43.00	59.00	116.00	243.00
0.07	77.37	73.71	6.00	10.00	24.00	55.00	107.00	174.00	223.00
0.08	69.78	67.48	5.00	9.00	22.00	50.00	96.00	157.00	203.00
0.09	64.72	62.68	5.00	9.00	21.00	46.00	88.00	146.00	189.00
0.10	61.12	59.14	5.00	8.00	19.00	43.00	84.00	138.00	179.00
0.40	2.92	0.32	2.00	3.00	3.00	3.00	3.00	3.00	3.00
1.00	1.00	0.00	1.00	1.00	1.00	1.00	1.00	1.00	1.00
2.00	1.00	0.00	1.00	1.00	1.00	1.00	1.00	1.00	1.00

**Table 6:** ARL and SDRL outcomes for comparative analysis.

Shift	SVM-EWMA at $\lambda$ = 0.10		RAEWMA at $\lambda$ = 0.10		SVM-EWMA at $\lambda$ = 0.25		RAEWMA at $\lambda$ = 0.25	
	ARL	SDRL	ARL	SDRL	ARL	SDRL	ARL	SDRL
0.00	370.35	372.60	370.58	361.57	370.01	369.13	369.23	366.99
0.01	247.61	246.79	300.25	301.65	195.62	193.49	344.28	342.51
0.02	160.03	153.45	248.92	243.01	135.28	133.99	320.51	316.52
0.03	102.39	95.84	204.31	196.66	121.95	118.82	292.93	291.14
0.04	78.81	71.76	168.99	162.36	104.12	100.73	271.29	268.45
0.05	64.56	59.69	143.23	133.30	84.76	82.26	250.15	248.66
0.06	56.61	50.40	118.56	109.42	75.74	74.15	226.14	220.77
0.07	47.49	40.13	104.15	95.62	70.03	67.46	212.31	211.26
0.08	40.82	34.14	86.33	78.53	63.86	61.48	196.40	192.95
0.09	36.00	29.14	75.58	66.27	64.76	62.35	183.39	180.39
0.10	30.87	23.50	65.89	57.43	61.28	59.57	168.10	163.27
0.40	2.91	0.33	9.80	5.23	6.00	4.33	10.79	7.27
1.00	1.0	0.0	3.47	1.13	1.00	0.00	2.86	1.16
2.00	1.0	0.0	2.05	0.33	1.00	0.00	1.37	0.54

## Conclusion

The application of statistical process monitoring tools extends far beyond the industrial sector and finds utility in diverse fields like healthcare. However, in healthcare, adjusting for risk factors is crucial prior to employing control charts. This study focuses on cardiac surgical data and emphasizes the necessity of adjusting patients' risk factors using an SVM model before implementing control charts. The newly proposed control chart in this study adapts the smoothing constant's value based on estimated shifts. The resulting ARL values across various shifts and different smoothing constants are meticulously presented in tables, underscoring the effectiveness of our proposed chart. This research highlights that our proposed SVM-EWMA control chart exhibits superior efficiency compared to its counterpart in monitoring healthcare processes. This emphasizes the importance of adapting statistical tools like control charts to suit the specific needs and nuances of healthcare contexts, particularly in assessing and managing risks associated with cardiac surgeries.

## Supplementary Information

Supplementary Information.

## Data Availability

The datasets used or analyzed in the ongoing study are held by the corresponding author, who can provide access to interested parties upon request. This procedure allows individuals seeking the data for further examination or validation to contact the corresponding author for access.
