# Supplementary material for: Risk adjusted EWMA control chart based on support vector machine with application to cardiac surgery data
Source: Sci Rep. 2024 Apr 26;14:9633. doi: 10.1038/s41598-024-60285-2 (PMC11584622; doi:10.1038/s41598-024-60285-2)
Supplement: Supplementary file 1 — Supplementary Information. [file 41598_2024_60285_MOESM1_ESM.docx]

**Appendix A**

**Model Training:**

1. **Data Preprocessing**:
   - The dataset underwent preprocessing, including the logarithmic transformation of survival times and designation of censoring indicators.
   - The covariates, such as the surgeon's identity and Parsonnet score were included in the dataset to account for potential variations in performance among surgeons over time.
2. **Training Data Selection**:
   - The training dataset consisted of Phase-I data spanning the initial two years of the study period.
3. **Model Training (SVM Regression)**:
   - The SVM regression model was trained using the **survivalsvm** package in R.
   - The survival time and censoring indicator served as response variables, while covariates such as the parsonnet score and surgeon identity were utilized as predictors.
   - Parameters such as the regularization parameter (**gamma.mu =1**), and radial basis function (RBF) kernel were specified during model training.
4. **Resultant Values**:
   - The trained SVM model was evaluated on the training set and predicted survival times were obtained using the **predict** function, and the errors were calculated.

The above steps allowed for the exploration of SVM regression as an alternative modeling approach to evaluate survival outcomes in cardiac surgery patients, accounting for the influence of covariates, including surgeon identity and parsonnet score.
